# The human milk endocannabinoidome and neonatal growth in gestational diabetes

**DOI:** 10.3389/fendo.2024.1415630

**Published:** 2024-06-13

**Authors:** Alice Fradet, Sophie Castonguay-Paradis, Camille Dugas, Julie Perron, Gabrielle St-Arnaud, Isabelle Marc, Alain Doyen, Nicolas Flamand, Fadil Dahhani, Vincenzo Di Marzo, Alain Veilleux, Julie Robitaille

**Affiliations:** ^1^ Centre NUTRISS - Nutrition, santé et société, INAF, Université Laval, Québec City, QC, Canada; ^2^ Ecole de nutrition, Faculté des sciences de l’agriculture et de l’alimentation (FSAA), Université Laval, Québec City, QC, Canada; ^3^ Canada Research Excellence Chair in the Microbiome-Endocannabinoidome Axis in Metabolic Health (CERC-MEND), Université Laval, Québec City, QC, Canada; ^4^ Département de pédiatrie, Université Laval, Centre de recherche du CHU de Québec, Québec City, QC, Canada; ^5^ Département des sciences des aliments, Faculté des sciences de l’agriculture et de l’alimentation (FSAA), Université Laval, Québec City, QC, Canada; ^6^ Centre de recherche de l’Institut universitaire de cardiologie et de pneumologie de Québec (IUCPQ), Université Laval, Québec City, QC, Canada; ^7^ Département de médecine, Faculté de médecine, Université Laval, Québec City, QC, Canada; ^8^ Joint International Unit on Chemical and Biomolecular Research on the Microbiome and its Impact on Metabolic Health and Nutrition (UMI-MicroMeNu), Pozzuoli, Italy

**Keywords:** gestational diabetes mellitus, human milk, mediators of endocannabinoidome, Npalmitoyl-ethanolamine (PEA), Weight-for-age z-score (WAZ)

## Abstract

**Objective:**

Endocannabinoids and their *N*-acyl-ethanolamines (NAEs) and 2monoacyl-glycerols (2-MAGs) congeners are involved in the central and peripheral regulation of energy homeostasis, they are present in human milk and are associated with obesity. Infants exposed *in utero* to gestational diabetes mellitus (GDM) are more likely to develop obesity. The objective of this cross-sectional study is to compare the profile of eCBome mediators in milk of women with gestational diabetes (GDM+) and without (GDM-) and to assess the association with offspring growth. The hypothesis is that the eCBome of GDM+ human milk is altered and associated with a difference in infant growth.

**Methods:**

Circulating eCBome mediators were measured by LC-MS/MS in human milk obtained at 2 months postpartum from GDM+ (n=24) and GDM- (n=29) women. Infant weight and height at 2 months were obtained from the child health record. Z-scores were calculated.

**Results:**

Circulating Npalmitoylethanolamine (PEA) was higher in human milk of GDM+ women than in GDM- women (4.9 ± 3.2 vs. 3.3 ± 1.7, p=0.04). Higher levels were also found for several 2monoacyl-glycerols (2-MAGs) (p<0.05). The levels of NAEs (β=-4.6, p=0.04) and especially non-omega-3 NAEs (B=-5.6, p=0.004) in human milk were negatively correlated with weight-for-age z-score of GDM+ offspring.

**Conclusion:**

The profile of eCBome mediators in human milk at 2 months postpartum was different in GDM+ compared to GDM- women and was associated with GDM+ offspring growth at 2 months.

**Clinical trial registration:**

ClinicalTrials.gov, identifier (NCT04263675 and NCT02872402).

## 1 Introduction

Gestational diabetes mellitus (GDM) is the most common medical complication in pregnant women and its prevalence has almost tripled from 24.7 to 75.5 per 1,000 births in 20 years in the province of Québec (Canada) (1). Children exposed *in utero* to GDM are at risk of obesity and type 2 diabetes in adulthood (2, 3). It has been shown that prolonged breastfeeding is beneficial, including protective effects against the development of obesity (4). However, there is a lack of knowledge regarding the impact of breastfeeding on infant health exposed *in utero* to GDM. This complication could lead to differences in the composition of human milk, as well as variations in satiety signals. Several studies have observed shorter breastfeeding duration in women with GDM (5, 6). Several factors can explain this reduced duration such as inadequate breastfeeding support (7), poorer sucking patterns (8) or lower lactogenesis for obese GDM women (9). Another explanation could also be differences in molecules linked to satiety signals in GDM+ breast milk (10). In turn, the regulation of appetite might be altered by milk composition, especially by bioactive lipid molecules known as endocannabinoid, and their congeners (11–14) which are recognized as being involved in energy metabolism in many tissues (15), and whose role in human milk biology is still poorly investigated. The endocannabinoid system includes the ligands, i.e., *N*arachidonoyl-ethanolamine (anandamide, AEA) and 2-arachidonoyl-glycerol (2AG), as well as the cannabinoid receptors (CB_1_ and CB_2_) and the metabolic enzymes of these mediators. The CB_1_ receptor is a key player in energy regulation. Its activation in the central nervous system increases food intake and suppresses anorexigenic signals (16). Similarly, activation of the CB_1_ receptor in the small intestine suppresses satiety signals following food intakes (17). Several congeners of AEA and 2-AG, especially the *N*acyl-ethanolamines (NAEs) and 2monoacylglycerols (2-MAGs) families, have been identified and now refer to as the endocannabinoidome (eCBome) (18). These congeners are ligands of various non-cannabinoid receptors also involved in the regulation of energy homeostasis and are associated with obesity and several metabolic complications (15, 19, 20) as well as with human behavioral parameters of food intake (21, 22). The eCBome mediators have been found in all tissues and biological fluids, including human milk (23, 24), but their precise role in human milk in health and diseases remain unknown. The objective of this study was to define the human milk eCBome profile in women who had a normoglycemic pregnancy or developed GDM during their pregnancy and assess its association with infant growth at 2 months of age. The hypothesis is that the eCBome of GDM+ human milk is altered and associated with a difference in infant growth.

## 2 Materials and methods

### 2.1 Study design and participants

For this cross-sectional study (NCT04263675), 30 women without GDM (GDM-) were recruited by email from *Université Laval* community between March to September 2020 and came for a single visit at the Institute of Nutrition and Functional Foods (INAF, Quebec, Canada). The inclusion criteria were as follow: no diagnosis of GDM, speak French fluently, singleton and term pregnancy (> 37 weeks), age ≥18 years and pre-pregnancy BMI ≥18.5 kg/m^2^. Exclusion criteria included multiple pregnancy, preterm delivery (< 37 weeks), a history of bariatric surgery or planning a pregnancy for the next year. Baseline data and samples of women with GDM (GDM+) who participated in an intervention study for 18 months, starting at two months postpartum were also used for this cross-sectional study. Women with a pregnancy complicated by GDM and followed by endocrinologists and dieticians at the two main hospitals with a neonatal care unit in Quebec City, Canada, were invited to participate in this clinical trial starting two months after delivery (NCT02872402). Invitations to participate in this study were also sent by emails to the community of *Université Laval*. The recruitment of 32 GDM+ women was conducted between January 2017 and September 2019. Same inclusion and exclusion criteria were used than for GDM- women except for the most recent pregnancy complicated by GDM for GDM+ women. Only baseline (2-month postpartum) data were used for this study. A total of 24 GDM+ and 29 GDM- women-infant pairs who had complete data at 2-month postpartum and human milk samples at this timepoint were included in this analysis (**Supplementary Material, Figure 1**). The population sample was pragmatically predetermined according to available funding. This study was conducted according to the guidelines laid down in the Declaration of Helsinki and all procedures were approved by the *Centre Hospitalier Universitaire de Québec* Ethics Committee (2017–3225 and 2020–5075).

### 2.2 Maternal data

During the 2-month postpartum visit, weight was measured on a calibrated balance to the nearest 0.1 kg, a stadiometer was used to measure the height and BMI was calculated (kg/m^2^). In the meantime, a dual energy X-ray absorptiometry scanner (DXA) measured body composition and body fat distribution. Fasting blood sample was collected for all women, and a 75 g 2-hour oral glucose tolerance test was performed. Plasma glucose at 0 and 2 hours were measured enzymatically and insulin at 0 and 2 hours were measured using the ADVIA Centaur CP Insulin (IRI) assay. This method is an *in vitro* diagnostic immunoassay for the quantitative determination of insulin in serum using direct chemiluminescent technology. Intra-assay CVs for low, intermediate, and high insulin concentrations were 4.6%, 3.2%, and 3.3%, respectively. Inter-assay CVs for low, intermediate, and high insulin concentrations were 5.9%, 2.6%, and 4.8%, respectively. Serum total cholesterol, triglyceride, and HDL cholesterol concentrations were assessed on a Roche/Hitachi Modular system (Roche Diagnostics). Serum LDL-cholesterol concentrations were calculated with the Friedewald equation (25).

### 2.3 Infant growth

Weight-for-age (WAZ), weight-for-length (WLZ) and length-for-age (LAZ) sex-specific z-scores at birth and at 2 months of age were calculated from weight and length using the growth standard charts from the World Health Organization (26). Infant growth between birth and two months, expressed as delta (Δ) values, were also calculated. Weight and length were obtained from the infant health record which includes measured weight and length from a health professional.

### 2.4 Human milk collection and processing

The day following the visit at 2 months postpartum, women were asked to collect 30 to 60 mL of human milk, at the end of a feeding, in a sterile cup where they recorded the date and time of the collection. Samples were stored in home freezers and brought frozen to the research center in a transport bag containing an ice pack. For consistency, all human milk samples were stored in the lab’s or participant’s -20°C freezers for exactly one month and then transferred in -80°C freezers. Milk samples were thawed once on ice, vortexed, aliquoted and stored at -80°C until batch analysis. The energy, triglyceride, lactose and protein content of human milk has been previously reported (27). Research assistants in charge of milk samples processing were not aware of the participants’ group allocation.

### 2.5 Circulating NAEs and 2-MAGs in human milk

As previously described, high-performance liquid chromatography coupled to tandem mass spectrometry (LCMS/MS) was used to measure levels of NAEs and 2-MAGs in 200uL in human milk sample (28). The methods quantified NAE mediators including: *N*palmitoyl–ethanolamine (PEA), *N*oleoyl–ethanolamine (OEA), *N*linoleoyl–ethanolamine (LEA), *N*-arachidonoyl-ethanolamine (AEA), *N*-eicosapentaenoyl-ethanolamine (EPEA), *N*-docosapentaenoyl-ethanolamine (DPEA) and *N*-docosahexaenoyl-ethanolamine (DHEA), as well as 2-MAGs mediators including 2-palmitoyl-glycerol (2-PG), 2-oleoyl-glycerol (2-OG), 2-linoleoyl-glycerol (2-LG), 2-arachidonoyl-glycerol (2-AG), 2-eicosapenaenoyl-glycerol (2-EPG), 2-docosapentaenoyl-glycerol (2-DPG) and 2-docosahexaenoyl-glycerol (2DHG). The data are presented as 2-MAGs but represent the combination of the 2- and 1(3)-isomers because 1(3)-isomers are most likely generated- from the former via acyl migration.

### 2.6 Statistical analyses

Student’s t-test, or Wilcoxon for non-parametric variables, was used to compare the GDM+ and GDM- groups. Multiple factor analysis (MFA) and principal component analysis (PCA) were performed using the FactoMineR and the factoextra R package (version 1.0.7). The groups of variables in the MFA models were adiposity [fat mass (kg), visceral adipose tissue volume (cm^3^), n=2 variables], glycemic profile [Insulin (mmol/L), fasting glycemia (mmol/L), HOMA-IR, n=3 variables], lipid profile (triglycerides, n=1 variable) and milk composition [proteins (g/100mL), triglycerides (g/100mL), lactose (g/100mL), n=3 variables). Moreover, levels of eCBome mediators were included in the NAEs (n=6 variables) and the 2-MAGs (n=7 variables) groups. Generalized linear model (GLM) was used to test the association between eCBome mediators groups in human milk and newborn growth, adjusted for baby’s birth weight, with univariate analyses. The p-values used to determine significance were the nominal p-values, and a p<0.05 was used to determine significance. All statistical analyses were conducted with R software version 2022.02.3.

## 3 Results

Characteristics of the mothers and infants are presented in **Table 1**. As shown previously by Dugas et al. with this cohort, age and fat mass percentage of mothers were higher in the GDM+ (n=24) group than the GDM- group (n=29) (27). None of the participants in the GDM+ or GDM- group had diabetes at the 2-month postpartum visit. Moreover, gestational age at birth was lower in the GDM+ group and the sex of the infant differed according to the GDM status (27). WAZ at 2 months was higher among GDM+ infants than GDM- infants. GDM+ women were also characterized by higher plasma triglyceride levels and cholesterol/HDLc ratio, and lower HDLc levels compared to GDM- women.

**Table 1:** Characteristics of mothers and infants in the GDM- and GDM+ group.

	GDM+ (n=24)	GDM- (n=29)	P value
Maternal			
Age (years)	33.6 ± 3.6	30.0 ± 3.1	**<0.001** ^**^
Height (m)	1.65 ± 0.04	1.64 ± 0.07	0.60^a^
Weight (Kg)	84.8 ± 19.7	74.6 ± 17.6	0.067
BMI (kg/m^2^)	31.2 ± 7.1	27.7 ± 5.9	0.07
Fat mass (%)	42.8 ± 6.8	38.0 ± 8.6	**0.04** ^*^
Fasting glucose (mmol/L)	5.04 ± 0.42	4.80 ± 0.31	0.05
Fasting insulin (pmol/L)	50.9 ± 20.5	51.0 ± 35.34	0.64^a^
Hba1c (%)	5.0 ± 4.0	5.0 ± 0.2	0.14^a^
Cholesterol (mmol/L)	5.24 ± 1.13	5.09 ± 1.04	0.62
Triglycerides (mmol/L)	1.15 ± 0.46	0.84 ± 0.40	0.007^*a^
Hdlc (mmol/L)	1.56 ± 0.29	1.74 ± 0.37	**0.04** ^*^
Ldlc (mmol/L)	3.15 ± 1.05	2.96 ± 0.90	0.423
Chol/hdlc	3.45 ± 0.94	3.02 ± 0.80	**0.05** ^*^
Infant			
Gestational age at birth (weeks)	38.6 ± 1.0	39.41 ± 1.1	**0.01** ^*^
Breastfeeding			0.22^*b^
Exclusive	22 (91.7%)	29 (100%)	
Non exclusive	2 (8.3%)	0 (0%)	
Delivery			0.84 **^b^**
Vaginal Birth	22 (91.7%)	27 (93.1%)	
Cesarean	2 (8.3%)	2 (6.9%)	
Sex			0.01^*b^
Boys	17 (74%)	12 (41%)	
Girls	6 (26%)	17 (59%)	
Length (cm)			
Birth	50 ± 4.17	50.70 ± 1.60	0.42
2 months	57.77 ± 2.95	57.63 ± 1.73	0.86
Weight (Kg)			
Birth	3.37 ± 0.34	3.34 ± 0.38	0.75
2 months	5.48 ± 0.78	5,06 ± 0.71	0.08
LAZ			
Birth	0.64 ± 0.91	0.68 ± 0.88	0.89
2 months	0.17 ± 1.07	0.04 ± 0.83	0.66
WAZ			
Birth	0.11 ± 0.69	0.10 ± 0.84	0.98
2 months	0.23 ± 0.72	-0.32 ± 0.88	**0.03** ^*^
WLZ			
Birth	-0.52 ± 1,42	-0,64 ± 1,26	0.79
2 months	0.04 ± 0.73	-0.33 ± 1,07	0.24

The data are means ± the standard deviation. * p value < 0.05 and ** p value < 0.005 by Student t test, ^a^Wilcoxon test or ^b^chi-squared test between group of GDM status. Significantly different values are in bold.

BMI, body mass index; Hdlc, hight density lipoproteins cholesterol; Hba1c, glycated hemoglobin; Ldlc, low density lipoproteins cholesterol; Chol/hdlc, cholesterol to Hdlc ratio; LAZ, length for age z-score; WAZ, weight for age z-score; WLZ, weight for length z-score.

### 3.1 Human milk endocannabinoidome mediators profile in GDM

**Figure 1:**
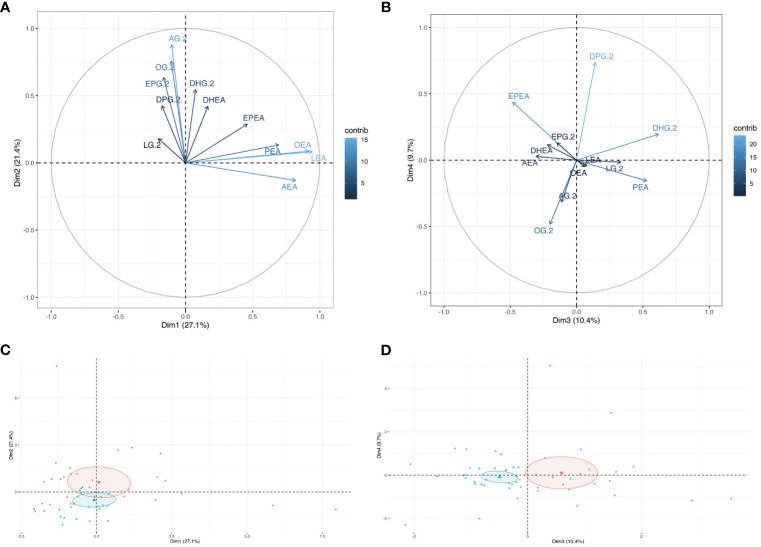
Visualization of the Principal component analysis (PCA) modeling the human milk endocannabinoidome. Graph of individuals of **(A)** dimensions 1 and 2, and of **(B)** dimensions 3 and 4 of the PCA model. Ellipses are confidence interval from the mean center of each group (GDM-: Red, GDM+: Blue). The Loading plot of all human milk NAEs and 2-MAGs in **(C)** dimension 1 and 2 and **(D)** dimension 3 and 4.

**Figure 2:**
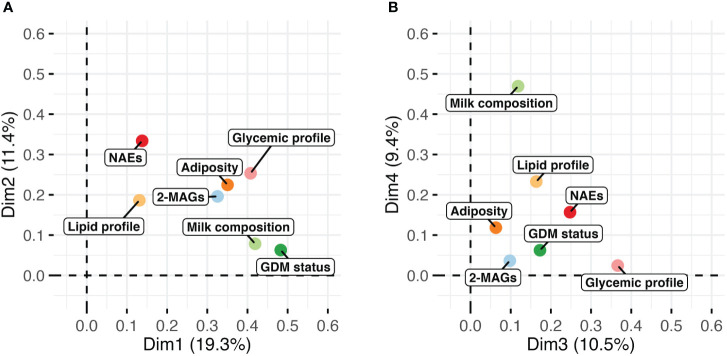
Visualization of the multiple factor analysis (MFA) modeling the milk endocannabinoidome with variables of adiposity (fat mass, visceral adipose tissue), glycemic (insulin, glycemia, HOMA-IR) and lipid (triglycerides) profile, milk composition (protein, lactose, triglycerides) and GDM status. Graph of factors contribution to **(A)** dimensions 1 and 2, and of **(B)** dimensions 3 and 4 of the MFA model.

Unsupervised analyses with human milk NAEs and 2-MAGs profiles were performed to determine whether the profile of eCBome mediators differed according to the GDM status (**Figure 1**). PCA analysis revealed that the profile of eCBome mediators in human milk was different between GDM+ and GDM- mothers. This difference was mainly clustered in the third dimension of the model (**Figure 1D**), of which human milk PEA, EPEA and 2-DHG levels were the main contributors (**Figure 1B**). We then computed a MFA model to explore the interrelation between GDM and the milk eCBome, while considering other variables (**Figure 2**). Adiposity of the mother (fat mass and visceral adipose tissue) was correlated with 2-MAGs in the human milk in the first and second dimension, these variables shared a significant part of the variance. Moreover, the first dimension of the MFA model also showed that GDM status, milk composition (protein, lactose, triglycerides), glycemic profile (Insulin, glycemia, HOMA-IR) and milk 2-MAG levels shared a significant part of the variance (**Figure 2A**). Milk NAE levels and the glycemic profiles were sharing variance with adiposity in the second dimension (**Figure 2A**), and with GDM in the third dimension of the model (**Figure 2B**).

**Figure 3:**
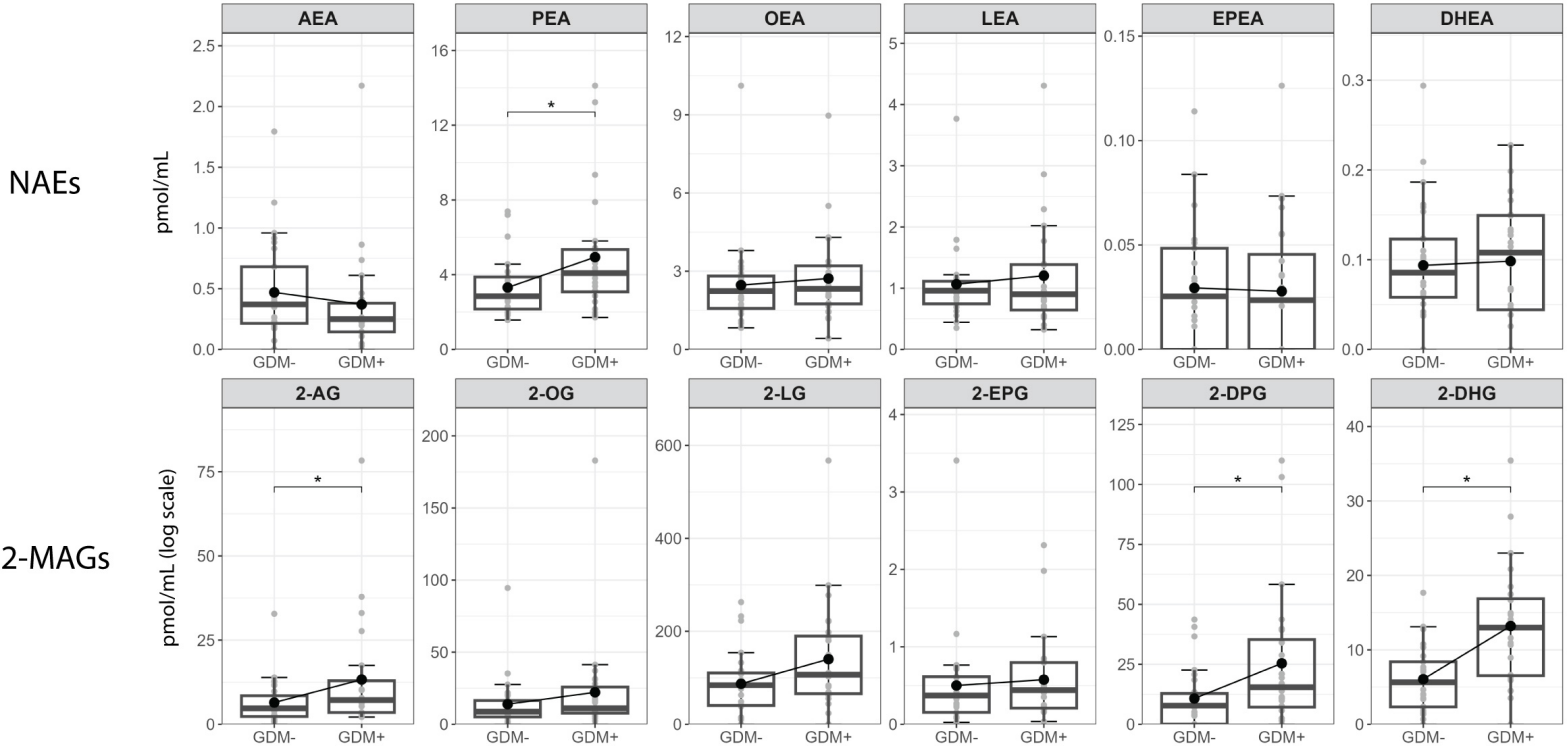
Human milk levels of NAEs and 2-MAGs according to GDM status. Boxplots include the median, lower/higher quartiles and 1.5x inter-quartile range whiskers. *Adjusted p < 0.05 (Wilcoxon sign rank test).

**Figure 3** shows the comparison between individual NAEs and 2-MAGs mediators in human milk of GDM- and GDM+ mothers. Within NAEs, we found significantly higher PEA levels in GDM+ human milk than in GDM- human milk. For 2-MAGs, the levels of 2-AG, 2-DPG and 2-DHG were higher in human milk from GDM+ women than GDM- women.

### 3.2 Human milk endocannabinoidome mediators profile and infant growth

Given the well-described association of eCBome mediators with the development of obesity and diverse metabolic complications, we examined whether there was a possible link between levels of eCBome mediators in human milk of GDM+ and GDM- women and infant growth (**Table 2**). Interestingly, in infants exposed *in utero* to GDM, lower human milk levels of NAEs, and more specifically of non-omega-3 derived NAE levels, were significantly negatively associated with WAZ and WLZ at 2 months, independent of weight at birth. Similarly, LAZ at 2 months was negatively associated with human milk levels of non-omega-3 derived NAEs in the entire study sample and among GDM+ women. Of note, while we observed an association between the GDM status and the levels of 2-MAGs in human milk, there was no significant association between levels of 2-MAGs in human milk and infant growth at 2 months, i.e., WAZ, LAZ and WLZ.

**Table 2:** GLM linear regression analyses modeling infant growth with the levels of endocannabinoidome mediators in human milk, adjusted for birth weight.

	Total group		GDM+		GDM-	
	Estimate	Pr > |t|	Estimate	Pr > |t|	Estimate	Pr > |t|
WAZ 2-months						
All NAEs	-0.8	0.54	**-4.64**	0.04*	0.73	0.63
non-omega 3 NAEs	-1.43	0.23	**-5.58**	0.004**	0.37	0.79
omega 3 NAEs	0.58	0.55	-0.06	0.96	0.94	0.48
All MAGs	1.36	0.37	-1.54	0.44	3.91	0.32
non-omega 3 MAGs	0.72	0.52	-0.8	0.59	0.32	0.87
omega 3 MAGs	1.01	0.45	-1.34	0.44	2.63	0.36
LAZ 2-months						
All NAEs	-3.07	0.06	-4.95	0.13	-2.02	0.26
non-omega 3 NAEs	**-3.66**	0.01*	**-5.73**	0.04*	-2.43	0.13
omega 3 NAEs	-0.01	0.99	-0.05	0.98	0.03	0.98
All MAGs	0.44	0.82	-0.54	0.86	4.69	0.23
non-omega 3 MAGs	0.6	0.67	-1.07	0.64	4.17	0.07
omega 3 MAGs	1.25	0.46	0.45	0.87	4.74	0.1
WLZ 2-months						
All NAEs	0.79	0.66	**-5.46**	0.03*	3.64	0.14
non-omega 3 NAEs	0.7	0.67	**-5.5**	0.01*	3.61	0.1
omega 3 NAEs	0.29	0.83	-0.98	0.52	1.31	0.55
All MAGs	0.87	0.59	-0.34	0.87	0.34	0.95
non-omega 3 MAGs	-0.03	0.98	0.73	0.66	-4.79	0.12
omega 3 MAGs	-0.6	0.68	-1.5	0.38	-2.28	0.54

Analyses were performed between eCBome mediators groups in human milk and newborn growth, adjusted for birth weight, with univariate analyses. * p value < 0.05, ** p value < 0.005. Significantly different values ​​are in bold.

## 4 Discussion

This study showed that a pregnancy complicated by GDM is associated with the profile of endocannabinoids and their congeners, i.e., the eCBome mediators, in human milk. To our knowledge, this is the first time that this comparison in the milk content of GDM+ and GDM- women is performed although previous studies have observed the presence of eCBome mediators in human milk (23, 29). Noteworthy, 1) milk of GDM+ women were characterized by significantly higher concentrations of PEA, 2-AG 2-DPG 2-DHG; and 2) changes in the human milk eCBome in the presence of GDM were associated with infant growth independently of weight at birth. Indeed, human milk non-omega-3 derived NAEs were negatively associated with WAZ and WLZ at 2 months in infants exposed to GDM *in utero*.

Results of this study suggest the presence of an alteration in the milk eCBome profile following a pregnancy with GDM. Indeed, we observed that the eCBome profile in human milk was altered in the early postpartum period as PEA and some 2-MAG congeners, but not AEA, were elevated in GDM+ women compared to GDM- women. It has been previously reported that pregnant GDM+ women have higher circulating levels of AEA and 2-AG than pregnant GDM- women (30) and that adiposity has been positively associated with circulating eCBome profile (31). Accordingly, we observed that levels of mediators of the human milk eCBome were also correlated with maternal adiposity (**Figure 2A**) suggesting that higher levels in GDM+ may be explained by the higher rates of adiposity observed among GDM+ women. Of the elevated eCBome mediators, 2-AG is recognized as an endogenous CB_1_ receptor agonist and thus modulates energy metabolism by increasing appetite and taste for palatable food (32). Therefore, its higher levels in milk, as a signal previously associated with suckling in mouse pups (12), may have contributed to the higher growth observed in GDM+ infants. However, the two other 2-MAG congeners, i.e., 2-DPG 2-DHG, were also higher in the milk of GDM+ mothers, and, since these compounds are less studied than 2-AG, conclusions regarding their exact metabolic roles cannot be made (21). It is possible that the higher levels of 2-MAGs observed in GDM+ milk samples are the results of increased levels of triglycerides found in these samples (27), which may act as 2-MAG precursors.

Also, although at birth, WAZ was similar between newborns exposed or not to GDM *in utero*, at 2 months of age, infants exposed to GDM *in utero* had higher WAZ than infants without GDM exposure. Higher rates of childhood obesity have been previously reported in a large follow-up cohort among infants exposed *in utero* to hyperglycemia, such as GDM (33). The higher weight observed among infants exposed to GDM *in utero* could be partly related to the composition of human milk as these differences in weight were not observed at birth. However, an interesting negative association was observed between non-omega-3 derived NAEs, including PEA, and infant WAZ at 2 months. To our knowledge, these results are novel as the characterization of human milk eCBome in this population has not been performed before. PEA supplementation has been shown to reduce body fat in mice, and high levels of PEA were shown to promote the browning of white adipose tissue (34). This is also true for OEA and LEA, which by binding to various receptors, such as peroxisome proliferator-activated receptor (PPAR)-α, orphan G-protein-coupled receptor 119 (GPR119) and transient receptor potential of vanilloid type-1 (TRPV1) channels, were also shown to reduce food intake through central and peripheral mechanisms (35). It could be possible that higher PEA (and 2-DPG) levels may act to prevent infants from gaining too much weight. Therefore, higher human milk PEA levels following GDM may represent an adaptative response to reduced food intake to counterbalance long-term GDM consequences in infants.

It should also be noted that the eCBome system is active from birth. The CB receptors and the AEA and 2-AG mediators have been shown to be expressed in the fetus, well before birth, and their expression is maintained after birth (36). Whether human milk eCBome mediators can travel to the brain and impact energy metabolism directly is not clear, but they could impact the brain via the brain-gut axis and play a role in the energy metabolism of the intestine (37). Moreover, previous studies in rodents have shown that endocannabinoid/CB1 signaling pathway plays a key role in suckling only during the very first days after birth in mice (12), and that rat pups with lower endocannabinoid levels, born from under-nourished dams, do not gain as much body weight as control pups until the end of lactation (38). Thus, based on that later study and our present results, more prolonged breastfeeding following GDM might be a way to allow non-CB1-mediated (and anorexigenic) eCBome signaling to prevail on CB1-mediated (and orexigenic) endocannabinoid signaling from milk, and could remain a good option to alleviate the negative impact of GDM on the infant future health. However, more studies will be needed to understand the role of the eCBome mediators in human milk as well as the mechanisms linking these mediators to GDM and infant growth. It must be emphasized that results from this study do not allow to conclude that changes in the milk composition in eCBome mediators are the only cause of increased body weight in infants from GDM+ mothers.

We acknowledge some study limitations, as we collected and analyzed milk samples only at 2-month postpartum within a small sample size. It would have been interesting to follow the evolution of eCBome mediators profile in the milk as well as the growth of the infant over the following months. Moreover, volume of milk produced and consumed by the infants was not collected which would have provided relevant information on milk secretion and infant satiety according to GDM status. Finally, the results of this study cannot be generalized to other populations as the study cohort includes mainly Caucasian women with a high degree of education. Therefore, additional studies with larger sample size are needed. However, this study had several strengths despite these limitations. Indeed, given the role of the eCBome profile in obesity and the association between human milk and reduced obesity risk, this study is the first, to our knowledge, to assess the association of the human milk eCBome profile with GDM and to study the association of the human milk eCBome profile with growth among infant exposed *in utero* to GDM which are at increased risk of childhood obesity. Also, strengths of this study include simultaneous collection of human milk samples with infant growth measures as well as with maternal adiposity and cardiometabolic profile during the 2-month postpartum visit. In addition, milk samples processing was blinded to research assistant which limits information bias.

## 5 Conclusions

Results of this study suggest that the profile of eCBome mediators in human milk is altered following GDM. Higher levels of PEA were found in the milk of women with GDM, and the levels of NAEs non-omega-3 (including PEA) were negatively correlated with infant growth. Additionally, although not correlated with infant growth indicators, the levels of 2-MAGs were also increased in GDM+ milk. This study is the first to report an independent association between human milk eCBome mediators and the growth of infants in the context of GDM. Mechanistic studies are needed to identify the mechanisms behind this association. Nevertheless, these results suggest that both maladaptive and adaptive eCBome-mediated responses may exist in human milk from GDM+ mothers, and that the former ones might prevail during breastfeeding, and be associated with infant weight at 2 months.

## Data availability statement

The raw data supporting the conclusions of this article will be made available by the authors, without undue reservation.

## Ethics statement

The studies involving humans were approved by Comité d’éthique de la recherche du CHU de Québec-Université Laval (CHU). The studies were conducted in accordance with the local legislation and institutional requirements. Written informed consent for participation in this study was provided by the participants’ legal guardians/next of kin.

## Author contributions

AF: Writing – original draft, Writing – review & editing, Formal analysis. SC-P: Writing – review & editing, Formal analysis. CD: Writing – review & editing, Investigation. JP: Writing – review & editing, Project administration. GS-A: Writing – review & editing, Investigation. IM: Writing – review & editing. AD: Writing – review & editing. NF: Writing – review & editing. FD: Writing – review & editing. VD: Writing – review & editing. AV: Writing – review & editing. JR: Writing – original draft, Writing – review & editing, Conceptualization, Funding acquisition, Supervision.
