# Supplementary material for: The human milk endocannabinoidome and neonatal growth in gestational diabetes
Source: Front Endocrinol (Lausanne). 2024 Jun 13;15:1415630. doi: 10.3389/fendo.2024.1415630 (PMC11208692; doi:10.3389/fendo.2024.1415630)
Supplement: Supplementary file 1 [file DataSheet_1.docx]

A

B

**Figure 1**. Participants flowcharts. A) for GDM- group. B) for GDM+ group
